# Nutrition education in Southeast Sulawesi Province, Indonesia: A cluster randomized controlled study

**DOI:** 10.1111/mcn.13030

**Published:** 2020-05-28

**Authors:** Devi Savitri Effendy, Pattaneeya Prangthip, Ngamphol Soonthornworasiri, Pattanee Winichagoon, Karunee Kwanbunjan

**Affiliations:** ^1^ Department of Tropical Nutrition and Food Science, Faculty of Tropical Medicine Mahidol University Bangkok Thailand; ^2^ Department of Public Health, Public Health Faculty Halu Oleo University Kendari Indonesia; ^3^ Department of Tropical Hygiene, Faculty of Tropical Medicine Mahidol University Bangkok Thailand; ^4^ Institute of Nutrition Mahidol University Bangkok Thailand

**Keywords:** child feeding practice, dietary diversity, Indonesia, nutrition education, nutritional status, randomized controlled study, Southeast Sulawesi

## Abstract

This study evaluated the impact of a nutrition education intervention on child feeding practices and children's nutritional status. Using a randomized controlled trial, we conducted an intervention for 6 months among caregivers with children aged 6–17 months in two subdistricts of Kendari, SE Sulawesi Province, Indonesia. In all, 22 integrated health posts were randomly assigned to an educational intervention or control group with 266 participants in both groups. Participants in the intervention group attended four nutrition classes and received a monthly home visit by cadres (community volunteers), whereas participants in the control group only received standard monthly health care at the health post. The primary study outcome was children's dietary diversity scores (DDSs). Mixed model analysis was conducted to examine the intervention effects on DDS and children's growth adjusting for clustering within subvillages. The study showed the educational intervention had a significant effect on children's DDS. Children in the intervention group had a larger DDS compared with children in the control group (Beta [mean difference] = 0.34, 95% CI: 0.02 to 0.66, *P* = 0.038). The intervention effect on height‐for‐age z‐score (HAZ) could not be shown (Beta = 0.24, 95% CI: −0.06 to 0.56, *P* = 0.112). However, stunting prevalence remained stable in the intervention group but increased in the control group. These results indicated nutrition education delivered through nutrition classes combined with regular home visits by cadres as influencers provided a great potential to be adopted to complement other nutrition programmes in community health centres.

**Key Messages**

- Nutrition education conducted through nutrition classes combined with regular home visits to promote diverse diets using local food can effectively improve child feeding practices concerning dietary diversity. These approaches provide a great potential to be adopted as part of nutrition programmes in all community health centres in Kendari, SE Sulawesi.
- Using *Posyandu*'s cadres as influencers during the home visits successfully encouraged the participants' to follow the educational intervention.
- Nutrition education in the community should stress more on consuming eggs and nuts as protein sources and the importance of continued breastfeeding to support child growth until 2 years.

## 1 INTRODUCTION

In Indonesia, child stunting remains a significant problem and raises serious concerns because of its high prevalence. Indonesia Basic Health Research (IBHR) reported that stunting prevalence in the last 10 years was from 30% to 37%, indicating stagnant progress. Twenty provinces in Indonesia showed a stunting prevalence higher than the national average. SE Sulawesi Province, where the present research was conducted, has been listed among the top 7 provinces in terms of the stunting rate, with a stunting prevalence of 42.6% in 2013 (Indonesia Health Ministry, 2007, 2010, 2013).

The complementary feeding period from 6 to 24 months of age is characterized by the highest nutrient requirements to support optimal child growth and development (Dewey, 2016). Insufficient quantities, less varied food types and low feeding frequency adversely affect children's nutritional status. In Indonesia, stunting and underweight prevalence among children aged 0–24 months has been shown to rise with increasing age (Julia, 2009), indicating a reflection of either poor nutrition during the complementary feeding period or other factors including infection, low maternal height and education, poor sanitation and hygiene and low socio‐economic status (Bardosono, Sastroamidjojo, & Lukito, 2007; Beal, Tumilowicz, Sutrisna, Izwardy, & Neufeld, 2018; Sandjaja et al., 2013; Torlesse, Cronin, Sebayang, & Nandy, 2016). Indonesia Food Consumption Survey in 2014 revealed deficient vegetables and fruits consumption among children under 5; the proportion of consumed vegetables was 48%, whereas fruits was under 10%. This survey reported that consumed animal food among children was also poor (<20%), even for seafood (Indonesia Health Ministry, 2014). Recent IBHR data reported that only 46.6% children aged 6–23 months met the World Health Organization's (WHO's) recommendation for minimum dietary diversity (MDD). The situation was worst in the age group 6–11 months as only 29.8% were fed according to recommendations (Indonesia Health Ministry, 2019).

Little is known about the factors related to stunting in SE Sulawesi, but poor feeding practices characterized by low quantity and quality of food during the vulnerable development stage are important factors associated with undernutrition here and in other regions of Indonesia. In 2018, IBHR reported that children aged 6–23 months achieving MDD totalled only 24.7% in this region (Indonesia Health Ministry, 2019). A systematic literature review on the potential determinants of child feeding practices among Indonesian children showed that at the individual level, lack of maternal nutrition knowledge, beliefs and perceptions was the barrier to achieving optimal feeding practices (Blaney, Februhartanty, & Sukotjo, 2015). A case control study in Konawe District in SE Sulawesi showed that mother's nutrition knowledge and child feeding practices were significantly associated with underweight status in children under five (Tasnim, Mwanri, & Dasvarma, 2018).

The Indonesian government provides basic health and nutrition services that reach communities from various economic statuses at subvillage levels through integrated health service posts (*Posyandu*). *Posyandu* is designed to provide five main services consisting of registration activity, weighing, recording the result of weighing, nutrition counselling and providing certain health services, such as immunizations, Vitamin A distribution and food supplementation. All services are conducted by health volunteers (cadre) except for the last services that provided by midwifes or nutrition officers. However, from our observation in the research location, nutrition counselling services were rarely provided. The limited attention to counselling efforts is also a problem for other *Posyandu* in Indonesia. This is related to a lack of or low knowledge levels and skills in nutritional counselling from cadres (Khomsan et al., 2014; Nazri et al., 2015) and limited service time to divert all activities on *Posyandu* Day (Februhartanty, 2005).

According to the Indonesian Ministry of Agriculture Report in 2018, Kendari in SE Sulawesi is categorized as a region with a high food security index reflecting the adequate availability and affordability of food (Food Security Agency, 2018). The research site is a coastal area with abundant seafood production and is located in the centre of SE Sulawesi where villages have no barrier accessing markets (BPS, 2019). Its environment supports dietary diversity for children. However, our formative research in the location revealed that mother's knowledge and skills in preparing complementary feeding were limited and only followed family and local community habits. Mothers commonly introduced a single dish such as empty porridge (rice porridge without additional foods), bananas or instant cereals to their children at complementary feeding time. Providing a single dish lasts until a child reaches 8 months or even continues until 1 year.

Mother's nutritional knowledge is a crucial factor influencing her ability to choose nutritious foods to feed their children and affecting her ability to manage the available resources to provide food. Empowering mothers through nutrition education was shown to be effective in addressing poor feeding practices (Kuchenbecker, Reinbott, Mtimuni, Krawinkel, & Jordan, 2017; Reinbott et al., 2016; Waswa, Jordan, Herrmann, Krawinkel, & Keding, 2015) and infant growth (Penny et al., 2005; Shi, Zhang, Wang, Caulfield, & Guyer, 2010; Vazir et al., 2013). As poor feeding practices are considered one of the barriers to optimizing children under two nutritional statuses in this area, efforts to provide mothers nutritional knowledge is required. Therefore, in this study, we delivered nutrition education by combining two approaches: (1) mother nutrition classes with simple and practical messages. In the mother nutrition classes, we promoted dietary diversity through combining seafood with vegetables that were locally available, affordable and part of the daily diet of local residents. (2) Home visits used cadres as agents to strengthen the nutrition messages. Cadres of *Posyandu* were expected to serve as potential influencers because cadres interact more with the community. We assumed that intervention groups receiving standard care from a *Posyandu* plus nutrition education would obtain better feeding practices with dietary diversity as the main outcome compared with control groups only receiving *Posyandu*'s standard care. Also, we examined the intervention impact on children's height‐for‐age z‐score (HAZ).

## 2 METHODS

### 2.1 Study sites

This study took place in Kendari, the capital city of SE Sulawesi in eastern Indonesia. The total population of this city is about 347,496 inhabitants, including about 34,626 children aged 0–4 years (BPS, 2015). The 10 subdistricts in Kendari have varying numbers of *Posyandu*, depending on the subdistrict's population size. Two subdistricts, West Kendari and Nambo, were purposely selected as the study sites. These sites were selected according to their characteristics, namely, an undernourished children problem, a high level of fishery production and availability and affordability of food in traditional markets to support children's diet diversity. In the study areas, nutrition officers and cadres in 29 *Posyandu* provided monthly health care for children under five (Kendari Health Office Report, 2017). Because seven *Posyandu* in two villages were previously exposed to a formative study, only 22 *Posyandu* were included in the intervention study.

### 2.2 Study design and participants

This study was conducted using a cluster randomized pretest–posttest control group design. Twenty‐two *Posyandu* from 14 villages of Nambo and West Kendari were randomly assigned to an intervention or control group. The participants in intervention groups received standard health and nutrition care plus nutrition education intervention, whereas the participants in control groups only received standard health and nutrition care from the *Posyandu.* Standard care for children under 2 in all *Posyandu* included child growth monitoring, nutrition counselling, basic immunizations, Vitamin A supplementation and fortified biscuit for underweight children.

The criteria to participate in this study were mothers or caregivers of children aged 6–17 months listed in the integrated health service post, signed informed written consent and residing in the research area. Exclusion criteria included severe stunting (HAZ < −3SD) or severe underweight status (weight‐for‐age z‐score [WAZ] < −3SD) among children or illiteracy of the mother. A minimum sample size of 126 per group was calculated with 95% confidence, 90% power, 1.5 as the standard deviation, 0.7 increments in child dietary diversity score (CDDS; taken from a related study in Cambodia; Reinbott et al., 2016) and the assumption of 30% loss to follow‐up. In total, 266 mother–child pairs from 22 *Posyandu* working areas were eligible and enrolled in this study.

### 2.3 Nutrition education

Before initiating the intervention, we conducted formative research in two villages in the research area using qualitative methods from March to April 2018. Using in‐depth interviews, focus group discussions and observations, we collected information about local child feeding practices. We also interviewed health officers and cadres about child health problems and the complementary feeding programme in their working area. Discussion with the public health office head and nutrition officers generated a recommendation to use the cadres as agents to achieve our intervention target.

We called our educational intervention, ‘Gerakan Ibu Cerdas Anak Sehat Bergizi Baik’ (GEN ASIK), meaning ‘Smart Mother for Healthy Children Movement’. The GEN ASIK nutrition education programme was provided using two approaches: nutrition classes and home visits. Topics covered in the nutrition classes were taken from the Infant and Child Feeding Counselling Package developed by the UN Children's Fund for Indonesia. Findings from the formative study guided us to provide nutrition messages that fit local needs. For example, considering constant availability and low prices, only two animal sources foods—fish and eggs—were selected to promote in this intervention. We found that mothers did not give particular food such as eggs for children under 1 year to avoid allergy, so we added food allergy topics as part of our curriculum. Also, we found that mothers had difficulty in determining the adequacy of the amount of food given to their child. Therefore, in advising the amount of food that should be given to children, we used a tablespoon as the standard to substitute plates or bowls. Topics and key messages can be found in Table 1.

**TABLE 1:** Topics in the nutrition class

Sessions	Topic^a^	Specific messages
1	Continue breastfeeding, seven food groups and the benefit of food for children's health and growth, and food allergy and feeding a sick child.	• Breastfeed your child until 2 years. • Give your child the best food available at home. • Your child can eat any food that family members eat. • Continue breastfeeding during illness. • Give fluids and food more frequently during illness.
2	Five principles of feeding for children:	
	Meal diversity	• Mixed *bubur* ^b^ or rice with at least the other three groups of foods: ‐ Add fish or eggs in children's meals daily. ‐ Add nuts such as *tempe*, green and red beans in your child's meal daily. ‐ Add vegetables such as *Moringa* leaf, spinach, pumpkin and carrots in your child's meal daily. • Give healthy snacks daily such as fruits (banana, papaya, mango, orange, watermelon and coconut meat), fortified biscuits and home‐made snacks. • Avoid giving sweets and salty snacks (processed food) to your child.
	Texture	• Gradually increase the texture following child's age: ‐ At 6–7 months, start with thick *bubur* ^b^. ‐ At 8–9 months, give your child mushed food/finger food. ‐ At 9–12 months, give your child chopped food/finger food. ‐ At 12 months, give your child family food.
	Meal quantity	• Increase the amount of food as the child grows: ‐ Age 6 months: 2 or 3 spoonfuls ‐ Age 7 months: 3 to 5 spoonfuls ‐ Age 8 months: 6–7 spoonfuls ‐ Age 9 months: 8 to 9 spoonfuls ‐ Age 10 months: 10 to 11 spoonfuls ‐ Age 11 months: 12 to 13 spoonfuls
	Meal frequency	• For breastfed child: ‐ At 6 months, start with twice daily and gradually increase the frequency following the increase of child's age. ‐ From 9 months, give your child the main meals 3 times daily and healthy snacks 1 or 2 times daily. • For nonbreastfed child: ‐ Give your child main meals 3 times and healthy snack 1 or 2 times.
	Responsive feeding	• Do not force feed and be patient when feeding your child until finishing all food.
	Hygiene	• Wash yours and child's hands before meals.
3	Cooking practice 1	• Demonstration of preparing child's meal containing at least 4 food groups from locally available foods • Make nutritious food from food available at home: ‐ Anchovy powder ‐ Anchovy biscuits
4	Cooking practice 2	Make nutritious snacks from food available at home: ‐ *Bagea* ^c^ cookie ‐ *Tempe* ^d^ stick ‐ Spinach cake

^a^ Taken from the Infant and Child Feeding Counselling Package developed by the United Nations Children's Fund for Indonesia.

^b^ Bubur is traditional rice porridge in Southeast Sulawesi.

^c^ Bagea is traditional cookie made from Sea urchin.

^d^ Tempe is a soy food with high protein content.

The nutrition classes were held June 2018 over four sessions. Nutrition classes consisted of education lessons with lecture method using pictures and video as media, discussion sessions to enhance participants' understanding and cooking practice. The principal researcher, in cooperation with a medical doctor and nutritionists, provided the lectures during the nutrition classes. Mothers in the intervention group attended four nutrition classes at a community venue for 2.5 to 3 h twice weekly for 2 weeks. Each participant was provided a handbook containing information on complementary feeding and food recipes. The handbook was simple, practical and easy to read because messages were arranged following age group.

At the cooking practices, mothers learned how to prepare meals containing four food groups and home‐made snacks with locally available food. The research location was a coastal area where seafoods, for example, anchovies and sea urchins, were affordable. Therefore, we demonstrated how to make anchovy powder, anchovy biscuits and sea urchin cookies (*bagea*). Participants also practiced making healthy snacks from spinach and *tempe.* At the end of the cooking sessions, mothers fed their children with the snacks, and we observed together whether the child liked it or not.

From July to October 2018, home visits were conducted for caregivers in each intervention group. These follow‐up visits, were conducted once monthly, aimed to reinforce the messages that had been received by the participants during the nutrition class and established healthy complementary feeding patterns. To monitor the adoption of recommendations for feeding practices, we created a 2‐day self‐reporting food record form for children's consumption. A monthly health report form was also provided for recording any illness experienced by the children.

A total of 11 cadres were chosen as change agents during the home visits, and each was responsible for one intervention subvillage comprising 10–15 participants. Cadres that were recruited for this study involved women having educational attainment at least senior high school, residing in the area they were responsible for, being active cadres based on the recommendations of the health officer and committed to being change agents during the home visits activity. The 11 cadres involved in this study had previously received training to provide health and nutrition services at the *Posyandu* and had at least 5 years of experience as volunteers encouraging and mobilizing the community to use the *Posyandu.* Before the intervention began, cadres received a 2‐day training on complementary feeding practice principles and preparing children's dishes to meet WHO standards using affordable, local food. These cadres were tasked to repeat key messages, encourage mothers to practice the recommendations and collect the food record forms and health report forms from the participants. The principal researcher conducted monthly meetings with cadres at the end of the month to refresh cadres on their tasks and share problems that they found during the home visits. At the meeting time, we determined the participants who needed a joint visit with the cadre and principal researcher the following month.

### 2.4 Collecting data at baseline and endline

Baseline data were collected in May 2018, and endline data were collected 6 months later. In each subvillage, mother–child pairs, selected from the health service monitoring book, were invited to health service posts. They were asked to provide their child growth monitoring books to verify their child's age. Before collecting any data, written informed consent was obtained from the mothers.

A total of 10 assistants, who obtained a bachelor's degree in public health promotion or nutrition, were recruited for the study. They were trained concerning anthropometric measurement and the interview techniques for 24‐h recall. When collecting data, the principal researcher supervised the research assistants in performing their tasks and provided feedback for the next data collection.

Data were collected including information about mother and child characteristics, sociodemographics and household backgrounds. Participants' economic status was determined based on monthly income (average cash earned monthly, not including household valuable assets, savings or aid). The original measure was collapsed in three income groups: Indonesian rupiah (IDR) less than 2,170,000; IDR 2,170,000–3,000,000; IDR more than 3,000,000. Families in the top category received income below monthly regional minimum wage in the research area. The Indonesian government sets the monthly regional wage in SE Sulawesi at IDR 2,170,000 or equivalent to 155.24 USD monthly (BPS, 2019). Household food security was assessed using the US Household Food Security Survey Module (Melgar‐Quinonez et al., 2006).

Feeding practice indicators included children's dietary diversity scores (DDSs), MDD, minimum meal frequency (MMF) and minimum acceptable diet (MAD), assessed following WHO guidelines. The primary outcome was DDS, calculated on the basis of seven food groups: (1) grains, roots and tubers; (2) legumes and nuts; (3) milk; (4) flesh food (meat and fish); (5) eggs; (6) Vitamin A‐rich fruits and vegetables; and (7) other fruits and vegetables. The DDS assesses how many food groups are included in the food consumed by children during the past day, with scores ranging from 0 to 7. MDD is defined as consuming foods from four or more food groups daily. For breastfeeding children, MMF is defined as being fed twice or more daily for children 6–8 months or at least 3 times daily for children aged 9–23 months. For nonbreastfeeding children, MMF is defined as being fed four times or more daily. MAD is defined as receiving the MAD apart from breast milk. For breastfeeding children, MAD is achieved when they reach both MDD and MMF. For nonbreastfeeding children, MAD is achieved when they receive two or more milk feedings daily, achieve at least the MDD not including milk feeding and meet the MMF. All these indicators were measured using 24‐h recall (WHO/UNICEF, 2010).

Anthropometric parameters were measured following standardized procedures. Children's weight was measured using an electronic weighing scale with 0.01‐kg accuracy, and the supine length was measured using a horizontal board with a head and sliding foot piece with 0.1‐cm accuracy. Mother's weight was measured using an electronic weighing scale that was precise to 0.1 kg, and height was measured to the nearest 0.1 cm using a microtoise. All measurements were taken twice, and all equipment were calibrated every measurement day. Children's height and weight were converted to HAZ and WAZ using the WHO Anthro Software (WHO, 2010). A cut‐off score of <−2 standard deviations for HAZ and WAZ was used to classify stunting and underweight status, respectively.

### 2.5 Statistical analysis

Descriptive analysis was performed to provide general information concerning the characteristic of the study population. Differences in subject characteristics and observed variables between intervention and the control groups were tested using the independent *t* test for continuous variables, the Mann–Whitney test for ordinal variables or nonnormal data distribution and the *χ* ^2^ test for nominal variables. Independent *t* test was used to compare score changes on dietary diversity and children anthropometry between control groups and intervention groups. Effect size was calculated for the mean score change between control and intervention group. Difference in score changes was divided by the control group standard deviation to determine effect size. The effect of the intervention on DDS as the main outcome was estimated by mixed effects models adjusting clustering within subvillages, child's age, parent's education, parent's occupation, number of children and income. We also used mixed effects models to measure other outcomes (HAZ, WAZ, length and weight) accounting for clustering within subvillages and baseline characteristics (parent's education, parent's occupation, child's age, child's sex, mother's height, number of children, birth weight and birth length). SPSS, Version 18.0 for Windows, was used for data analysis (SPSS Inc., Chicago, IL, USA).

### 2.6 Ethical considerations

This study had been approved by the Ethics Committee of the Faculty of Tropical Medicine, Mahidol University, Bangkok (MUTM 2018‐045‐01) and by the Ethics Research Committee of LPPM Halu Oleo University, Kendari, Southeast Sulawesi Province, Indonesia (336/UN29.20/PPM/2018). This study was conducted following the guidelines stated in the Declaration of Helsinki and other international guidelines for human research protection. The objectives of the research were described, and informed consent was sought from the mothers of the children before interviewing.

## 3 RESULTS

**FIGURE 1:**
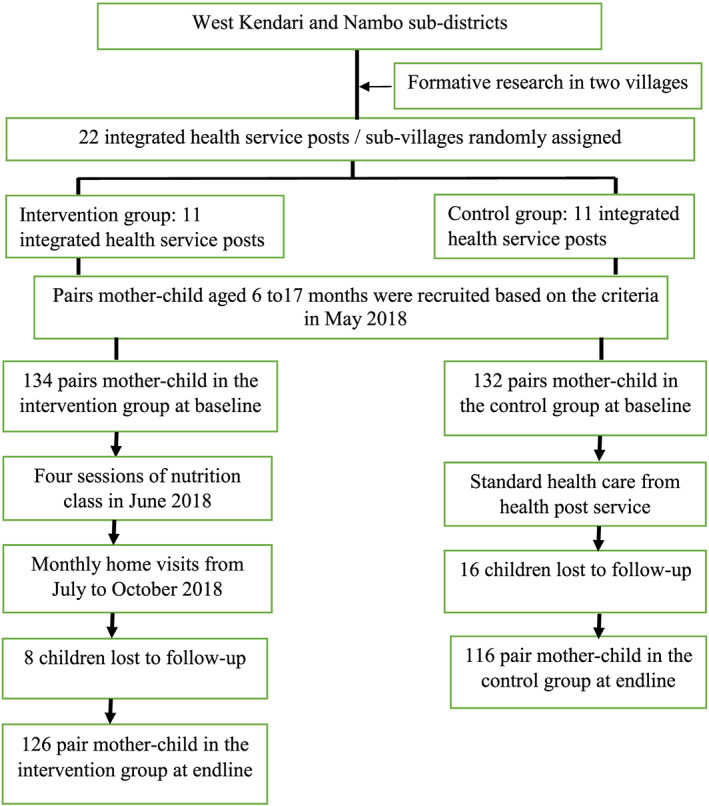
Trial profile

Figure 1 displays the trial profile. At baseline, 266 mother–child pairs were eligible to participate (134 pairs in the intervention and 132 pairs in the control groups). At endline, 242 mother–child pairs were available for final measurement (126 in the intervention and 116 in the control groups). Twenty‐four participants were unavailable because of migration or not being at home at follow‐up visit time.

Table 2 shows the characteristics of the study participants. No significant differences were found regarding any of the main characteristics between intervention and control groups.

**TABLE 2:** Study participants' characteristics comparing control and intervention groups, Kendari, SE Sulawesi Province, Indonesia

Characteristic	Control group^a^ (*n* = 116)		Intervention group^a^ (*n* = 126)		*P* value
	*n* (%)	Mean (±SD)	*n* (%)	Mean (±SD)	
Child's age (months)	‐	11.73 (±3.48)	‐	11.55 (±3.62)	0.700
Sex					0.830
Male	55 (47.4)	‐	58 (46.0)	‐	
Female	61 (52.6)	‐	68 (54.0)	‐	
Birth weight	‐	3.04 (±0.43)	‐	3.05 (±0.42)	0.859
Birth length	‐	48.27 (±1.49)	‐	48.5 (±1.54)	0.085
Exclusive breastfeeding					
For 3 months	77 (66.4)	‐	74 (58.7)	‐	0.220
For 6 months	4 (3.40)	‐	12 (9.50)	‐	0.057
Mother's age	‐	28.18 (±5.60)	‐	29.37 (±5.32)	0.091
Mother's height	‐	1.53 (±0.05)	‐	1.53 (±0.04)	0.827
Mother's weight	‐	52.97 (±10.37)	‐	54.16(±10.37)	0.374
Number of children	‐	2.41 (±0.95)	‐	2.61 (±0.89)	0.069
Mother's education					0.377
Elementary school	27 (23.3)	‐	21 (16.7)	‐	
Junior high school	28 (24.1)	‐	33 (26.1)	‐	
Senior high school	58 (50.0)	‐	70 (55.6)	‐	
University	3 (2.6)	‐	2 (1.6)	‐	
Mother occupation					0.241
Housewife	93 (80.2)	‐	93 (73.8)	‐	
Working outside	23 (19.8)	‐	33 (26.2)	‐	
Father's education					0.983
Elementary school	26 (22.4)	‐	19 (15.1)	‐	
Junior high school	25 (21.6)	‐	35 (27.8)	‐	
Senior high school	51 (44.0)	‐	66 (52.4)	‐	
University	14 (12.1)	‐	6 (4.8)	‐	
Father's occupation					0.635
Government	18 (15.5)	‐	27 (21.4)	‐	
Business	38 (32.8)	‐	41 (32.5)	‐	
Private/factories	16 (13.8)	‐	17 (13.5)	‐	
Casual labour	8 (6.9)	‐	11 (8.7)	‐	
Fishing	36 (31.0)	‐	30 (23.8)	‐	
Type of floor					0.129
Natural (ground/sand)	3 (2.6)	‐	2 (1.6)	‐	
Rudimentary (wood)	22 (19.0)	‐	13 (10.3)	‐	
Finished floor	91 (78.4)	‐	111 (88.1)	‐	
Type of roof					0.948
Thatch	3 (2.6)	‐	4 (3.2)	‐	
Tin	108 (93.1)	‐	116 (92.1)	‐	
Concrete	5 (4.3)	‐	6 (4.8)	‐	
Fuel for cooking					0.180
Kerosen	13 (11.2)	‐	8 (6.3)	‐	
LPG	103 (88.8)	‐	118 (93.7)	‐	
Source of drinking water					0.605
Government facility	20 (17.2)	‐	28 (22.2)	‐	
Wheel	29 (25.0)	‐	28 (22.2)	‐	
Galon water refill	67 (57.8)	‐	70 (55.6)	‐	
Having toilet facility at home	96 (82.8)	‐	111 (88.1)	‐	0.238
Monthly income (IDR)^b^					0.286
<2,170,000 IDR	24 (20.7)	‐	34 (27.0)		
2,170,000‐3,000,000 IDR	73 (62.9)	‐	74 (58.7)		
>3,000,000 IDR	19 (16.4)	‐	18 (14.3)		
Food security level					0.638
Food secure	83 (71.6)	‐	91 (72.2)	‐	
Moderately food insecure	12 (10.3)	‐	22 (17.5)	‐	
Severely food insecure	21 (18.1)	‐	13 (10.3)	‐	
Immunization status					0.505
Incomplete	31 (26.7)	‐	29 (23.0)	‐	
Complete	85 (73.3)	‐	97 (77.0)	‐	
Vitamin A supplementation					0.712
No	9 (10.2)	‐	11 (12.0)		
Yes	79 (89.8)	‐	81 (88.0)		

Abbreviations: IDR, Indonesian rupiah; SD, standard deviation.

^a^ Continuous variable written as Mean (±SD), categorical variable written as number.

^b^ Monthly income level is defined as average cash earned monthly, not including household valuable assets, savings or aid. The Indonesian government sets the monthly regional wage in SE Sulawesi at IDR 2,170,000 or equivalent to 155.24 USD monthly (BPS, 2019).

### 3.1 Coverage of standard health and nutrition care

Coverage of standard care was assessed by confirming the presence of children in the *Posyandu* through cadres' notes or checking the child's growth monitoring book. At baseline, data were only available for immunization and Vitamin A supplementation, and both showed no significance difference on the coverage. At endline, more children in the intervention group received Vitamin A supplementation compared with the control group (95.2 vs. 87.1%, *P* = 0.024). Percentage of mothers attended weighing session in August, September and October was higher in the intervention compared with the control group (87.3 vs. 71.6%, *P* = 0.002; 74.6 vs. 66.4%, *P* = 0.160; 80.2 vs. 40.5%, *P* = 0.000, respectively).

### 3.2 Child feeding practices

Table 3 presents child feeding practices at baseline and endline. No significant differences were observed for child feeding practices in intervention and control groups at baseline, except for continued breastfeeding (70.6% in the intervention vs. 56.0% in the control group, *P* = 0.018).

**TABLE 3:** Child feeding practices comparing baseline and endline data in Kendari, SE Sulawesi Province, Indonesia

	Control (*n* = 116)	Intervention (*n* = 126)	*P* value	Effect size (95% CI)
WHO indicator				
CDDS^a^				
Baseline	2.35 (±1.11)	2.37 (±1.11)	0.939	
Endline	3.32 (±1.33)	3.87 (±1.06)	0.001^*^	
Difference score	0.96 (±1.31)	1.49 (±1.36)	0.002^*^	0.40 (0.18 to 0.86)
MDD^b^				
Baseline	18 (15.5)	22 (17.5)	0.684	
Endline	56 (48.3)	88 (69.8)	0.001^*^	
MMF^b^				
Baseline	98 (84.5)	97 (77.0)	0.141	
Endline	98 (84.5)	108 (85.7)	0.788	
MAD^b^				
Baseline	14 (12.1)	14 (11.1)	0.816	
Endline	26 (22.4)	72 (57.1)	0.000^*^	
Currently breastfeeding^b^				
Baseline	65 (56.0)	89 (70.6)	0.018^*^	
Endline	60 (51.7)	76 (60.3)	0.178	
Food groups^b^				
Grains, roots, and tubers (group 1)				
Baseline	114 (98.3)	123 (97.6)	1.000	
Endline	113 (97.4)	125 (99.2)	0.352	
Legumes and nuts (group 2)				
Baseline	8 (6.9)	5 (4.0)	0.313	
Endline	9 (7.8)	36 (28.6)	0.000^*^	
Milk (group 3)				
Baseline	63 (54.3)	62 (49.2)	0.427	
Endline	59 (50.9)	53 (42.1)	0.170	
Flesh food (meat and fish) (group 4)				
Baseline	35 (30.2)	32 (25.4)	0.407	
Endline	76 (65.5)	102 (81.0)	0.007^*^	
Eggs (group 5)				
Baseline	20 (17.2)	20 (15.9)	0.775	
Endline	32 (27.6)	57 (45.2)	0.004^*^	
Vitamin A‐rich fruit and vegetables (group 6)				
Baseline	28 (24.1)	41 (32.5)	0.148	
Endline	66 (56.9)	92 (73.0)	0.009^*^	
Other fruits and vegetables (group 7)				
Baseline	5 (4.3)	16 (12.7)	0.021^*^	
Endline	30 (25.9)	22 (17.5)	0.112	
Consumption of selected food^b^				
Fruits				
Baseline	5 (4.3)	5 (4.0)	0.894	
Endline	6 (5.2)	38 (30.2)	0.000^*^	
Sweet and salty snacks (processing snack)				
Baseline	63 (54.3)	69 (54.8)	0.944	
Endline	76 (65.5)	57 (45.2)	0.002^*^	
Healthy snacks				
Baseline	19 (16.4)	18 (14.3)	0.651	
Endline	24 (20.7)	57 (45.2)	0.000^*^	

Abbreviations: CCDS, child dietary diversity score; CI, confidence interval; MAD, minimum acceptable diet; MDD, minimum meal frequency; SD, standard deviation; WHO, World Health Organization.

^a^ Continuous variable written as mean (±SD).

^b^ Categorical variable written as number (%).

* *P* < 0.05.

Children's dietary diversity was developed with a total score of 7. Between the 2 time points, mean DDS increased from 2.37 to 3.87 in the intervention group (Supporting information) and from 2.35 to 3.32 in the control group. The intervention group gained more points in diversity score than did the control group (1.49 ± 1.36 vs. 0.96 ± 1.31, *P* = 0.002). At endline, the percentage of children achieving two indicators, MDD and MAD, was significantly higher in the intervention compared with the control group (MDD: 69.8 vs. 48.3%; MAD: 57.1 vs. 22.4%). In terms of MMF, no significant change was found in both groups because it was high since baseline. We also observed a decrease in the percentage of mothers who continued breastfeeding in both groups.

The types of food consumed by children were grouped in seven food categories. After implementing the intervention, more children in the intervention than those in the control group consumed flesh foods (81.0 vs. 65.5%), eggs (45.2 vs. 27.6%), legumes and nuts (28.6 vs. 7.8%) and Vitamin A‐rich vegetables and fruits (73.0 vs. 56.9%). Moreover, more children in the intervention group than those in the control group reported consuming healthy snacks (45.2 vs. 20.7%). However, the percentage of children receiving sweets and salty snacks remained high in both groups (45.2 in the intervention and 65.5% in the control group). In terms of handwashing practice before preparing meals and feeding child, no significant difference was found in both groups (94.4 vs. 87.9%, *P* = 0.072; 92.9 vs. 88.8%, *P* = 0.272).

Further, the intervention effect on children's DDSs as the main outcome was analysed using a mixed model (Table 5). The result showed a positive significant intervention effect in which children in the intervention group showed a larger DDS than children in the control group (Beta = 0.34, *P* = 0.038, 95% confidence interval: 0.02 to 0.66).

### 3.3 Children's nutritional status

Table 4 presents the findings concerning children's nutritional status. At baseline, the anthropometry characteristics were similar in both groups. After implementing the intervention, increment in weight was significantly greater in the intervention than in the control group (1.19 ± 0.54 vs. 1.04 ± 0.53, *P* = 0.040), whereas increment in length did not significantly differ between the two groups (5.48 ± 1.21 vs. 5.27 ± 1.54, *P* = 0.261). From baseline to endline, a decreasing trend in mean HAZ scores was shown either in the intervention (−1.13 ± 1.00 to −1.52 ± 0.83) or control groups (−1.33 ± 1.08 to −1.73 ± 0.91). Change in mean HAZ scores did not have significant differences between the two groups (−0.38 ± 0.53 vs. −0.39 ± 0.64, *P* = 0.836).

**TABLE 4:** Children's anthropometry, stunting and underweight at baseline and endline in Kendari, SE Sulawesi Province, Indonesia

	Control (*n* = 116)	Intervention (*n* = 126)	*P* value	Effect size (95% CI)
Weight^a^				
Baseline	8.21 (±1.16)	8.19 (±1.19)	0.878	
Endline	9.26 (±1.25)	9.38 (±1.25)	0.460	
Difference score	1.04 (±0.53)	1.19 (±0.54)	0.040^*^	0.28 (0.006 to 0.279)
Length^a^				
Baseline	70.93 (±4.47)	71.14 (±4.43)	0.707	
Endline	76.21 (±4.26)	76.63 (±4.12)	0.439	
Difference score	5.27 (±1.54)	5.48 (±1.21)	0.261	0.13 (−0.15 to 0.55)
HAZ^a^				
Baseline	−1.33 (±1.08)	−1.13 (±1.00)	0.154	
Endline	−1.73 (±0.91)	−1.52 (±0.83)	0.067	
Difference score	−0.39 (±0.64)	−0.38 (±0.53)	0.836	0.01 (−0.13 to 0.16)
WAZ^a^				
Baseline	−0.98 (±1.01)	−0.95 (±0.88)	0.838	
Endline	−1.13 (±0.97)	−0.99 (±0.86)	0.236	
Difference score	−0.15 (±0.53)	−0.03 (±0.52)	0.093	0.22 (−0.01 to 0.25)
HAZ <−2 SD (stunting)^b^				
Baseline	33 (28.4)	25 (19.8)	0.117	
Endline	42 (36.2)	25 (19.8)	0.004^*^	
WAZ <−2 SD (underweight)^b^				
Baseline	24 (20.7)	18 (14.3)	0.189	
Endline	19 (16.4)	9 (7.1)	0.025^*^	

Abbreviations: CI, confident interval; HAZ, height‐for‐age z‐scores; SD: standard deviation; WAZ, weight‐for‐age z‐scores.

^a^ Continuous variable written as mean (±SD).

^b^ Categorical variable written as number (%).

^*^ *P* < 0.05.

From baseline to endline, stunting prevalence was observed to increase from 28.4% to 36.2% in the control group, whereas it remained stable (19.8%) in the intervention group. Moreover, both intervention and control groups showed a reduced prevalence of underweight (14.3% to 7.1% vs. 20.7% to 16.4%, respectively). At endline, stunting and underweight prevalence were significantly lower in the intervention than the control group (*P* = 0.004 and *P* = 0.025).

Using linear mixed model (Table 5), a positive but nonsignificant intervention effect was found concerning HAZ scores (Beta = 0.24, *P* = 0.112, 95% confidence interval: −0.06 to 0.56). In addition, no significant intervention effects of the nutrition education on WAZ scores, length and weight were observed.

**TABLE 5:** Intervention effect on children's dietary diversity scores (DDSs) and children's anthropometry using mixed model comparing intervention and control group

Variable^a^	Beta (95% confident interval)	*P* value
Dietary diversity scores^b^		
Intervention group	0.34 (0.02 to 0.66)	0.038*
Height‐for‐age z‐scores^c^		
Intervention group	0.24 (−0.06 to 0.56)	0.112
Weight‐for‐age z‐scores^c^		
Intervention group	0.04 (−0.26 to 0.35)	0.747
Length (cm)^d^		
Intervention group	0.59(−0.15 to 1.34)	0.112
Weight (kg)^d^		
Intervention group	0.03(−0.28 to 0.35)	0.825

^a^ All outcomes were adjusted for subvillages, parent's education, parent's occupation, income and number of children.

^b^ Includes child's age in the model.

^c^ Includes child's birth weight and child's birth length in the model.

^d^ Includes child's birth weight, child's birth length, child's age and child's sex in the model.

^*^ *P* < 0.05.

## 4 DISCUSSION

In this study, we promoted the importance of applying five principles of child feeding (meal diversity, meal quantity, meal frequency, meal consistency and hygiene). Meal diversity was the main focus in which mothers were encouraged to add fish and eggs as protein sources from animals, nuts, vegetables and fruits to their children's daily diets to reach a minimum of four of seven food groups, as recommended by the WHO. In this present study, information exposure through mothers' classes, strengthened by cadres' repeated home visits, improved the mothers' awareness of the importance of appropriate complementary feeding and subsequently increased the quality of children's diet. Simple and practical messages with explanations of the benefits of each food for children encouraged mothers to adopt new knowledge, which was reflected in the more diverse diet of children. A research on maternal nutrition knowledge and the demand for micronutrient‐rich foods in Indonesia found that mothers with nutrition knowledge allocated larger budget to buy micronutrient‐rich foods than did mothers without nutrition knowledge (Block, 2004). In this study, the intervention group exhibited higher achievement in diversity scores than those of the control groups, indicating the quality of children's diets increased in the intervention group. Moreover, a significant improvement was observed regarding two indicators of feeding practices, MDD and MAD, as shown in the intervention group. More children in the intervention group achieved MDD and MAD than those in the control group at endline. For MMF indicator, the proportion of children achieving in both groups already had been high since the baseline survey and did not change much afterward.

Our nutrition education provided new knowledge and information that overcame mother's incorrect feeding practice. Caregivers in the intervention group were more aware of the dangers of excessively consuming unhealthy snacks than at baseline, which could be seen in the decreased percentage of children consuming sweet and salty snacks, along with increased intake of healthy snacks. Our 24‐h recall showed between the two main meals, mothers in the intervention group provided their children with snacks such as local fruits, fortified biscuits and local cakes, either self‐made or purchased, mostly made from flour, coconut, bananas and eggs.

The success of several nutrition education interventions targeting improved complementary feeding practices has been well documented. Related nutrition education intervention programmes in China and Kenya, using similar approaches to those in this study (combining group training sessions, cooking demonstrations and home visits), were demonstrated to improve children's dietary diversity (Shi et al., 2010; Waswa et al., 2015). Likewise, interventions in Zimbabwe, Cambodia and Malawi showed improved quality of children's diets (Kuchenbecker et al., 2017; Paul et al., 2012; Reinbott et al., 2016). In Indonesia, a study in East Nusa Tenggara Province reported that educational intervention concerning mothers of children aged 9–16 months could improve children's nutrient intake. In this study, messages, developed using linear programming, were delivered once monthly for 6 months by the study team with voluntary health workers as assistants (Fahmida et al., 2015).

In this study, significant improvement of children's DDS in the intervention group was shown. Notably, mean CDDS remained below WHO minimum standard. For two recommended food groups, eggs and nuts, the percentage of children consuming these items remained lower than expected (<50%). Moreover, although the importance of continued breastfeeding was included as one of the nutrition messages and stressed in repeated home visits by cadres, the significant decrease was shown in the percentage of those still breastfeeding children. The main reason that prompted mothers to stop breastfeeding was low breast milk production (60%). Other reasons were pregnancy, the child wanted to stop and being busier. In this study, to avoid dissemination of complicated messages to participants as our main concern was improving the more diverse diet on children, we only inserted short explanations about the benefits of continued breastfeeding for children and mothers to support continued breastfeeding. For future interventions, messages to continue breastfeeding should be strengthened by providing problem solving in breastfeeding.

In the present study, HAZ decreased in both groups at endline, resulting in a nonsignificant treatment effect. However, the prevalence of stunting remained stable in the intervention group but increased in the control group. Low stunting prevalence in the intervention group might have been explained by improvements in consuming growth‐promoting, nutrient‐rich foods such as flesh food particularly fish and seafood, eggs and Vitamin A‐rich vegetables. A study conducted in Cambodia showed that consuming animal source food reduced stunting prevalence (Darapheak, Takano, Kizuki, Nakamura, & Seino, 2013). Moreover, reinforcing the cadres to increase protective caregiver behaviours may have affected outcomes. Significantly, the rate of children receiving Vitamin A supplementation and mothers attending weighing sessions was higher in the intervention compared with control group. The increase in stunting prevalence in the control group represents a warning signalling the need for more effective nutritional programmes to prevent stunting. The approaches we used in this educational intervention were promising as a solution to stunting problems in this area.

Several factors may have contributed to the lack of intervention effects on HAZ. First, the 5 months of education activity followed by evaluation in 6 months may have been too short to demonstrate an impact on child growth. By comparison, in China, significant effects of an educational intervention on WAZ, WHZ and HAZ were shown after an 18‐month follow‐up period (Zhang, Shi, Chen, Wang, & Wang, 2013). Another study in Peru also showed effects on WAZ and HAZ after an 18‐month follow‐up period (Penny et al., 2005). Second, breastfeeding declined in the intervention group at endline. This may have directly influenced children's growth. Breastfeeding was strongly, positively related to children's growth in that it continues to provide nutrients necessary during the second phase of life (Onyango, Esrey, & Kramer, 1999). Third, consuming unhealthy snacks remained relatively high (45%) in the intervention group. Snacking may have reduced children's appetite for their main meals, thus reducing their overall food consumption.

The main strength of our study was the use of *Posyandu* cadres as change agent to reach the target population. Cadres resided in the same villages as those of the study participants, and they knew the participants in their working area personally. This simplified the process of making repeated visits to participants' homes. In our study, we observed that cadres were a trusted source of information for mothers and they could be a connecting bridge between health services and the community. The strategy of enlisting the assistance of one cadre per subvillage as an influencer successfully encouraged participants. Participation was high in that all participants in the intervention group attended all nutrition class sessions and cooperated in the follow‐up visits. Less than 10% of participants were lost to follow‐up, and most participants were in the control subvillages. Another strength of our study was its randomized controlled design, its trained team and its use of standardized evaluation procedures.Some limitations were observed in our study. Due to the limited number of nutrition officers from the local health department, they were not directly involved in implementing educational interventions. Nutrition classes were delivered by a study team and may have affected nutrition class sustainability. For future interventions, involving nutrition officers and midwives in the nutrition education sessions would be important as they are the primarily responsible for health and nutrition care in their working area. Furthermore, in training the cadres, we focused on providing knowledge and skills concerning appropriate complementary feeding and gave minor concern to communication skills as an influencer during home visits. For future interventions, cadre training should include communication skills in the curriculum. Another limitation was our sample size calculation did not take clustering into account. We calculated the design effect (DE) by assuming the intercluster variation to be low at 0.01 (Dreyhaupt, Mayer, Keis, Öchsner, & Muche, 2017; Shi et al., 2010). With DE of 1.1, sample size gave 97% power to detect the 0.7 difference in CDDS as set previously. If there was no DE, the original sample size (126 per group) gave 95% power to detect the same difference; thus, it still gave enough power. Moreover, we only used of 24‐h recall to measure primary outcomes without another method to validate it. However, to increase the accuracy of mothers' reports concerning their children's diet, our field workers conducting interviews delivered expertise in interviewing using 24‐h recall method. Moreover, all interviewers were women with the same cultural background as participants making it easier for mothers to report the children's actual diet. Moreover, we did not record the quantity of children consumed. Thus, available data could not be further analysed. Future interventions should measure the amount of food that children consume to calculate nutrient intake in detail.

## CONFLICT OF INTEREST

The authors declare that they have no conflicts of interest.

## CONTRIBUTIONS

DSE, KK, PP, NS and PW designed the research. DSE performed the research, analysed the data and wrote the manuscript. KK supervised the research. KK and PW contributed to the critical revision of the manuscript. NS contributed to statistical analysis. All authors read and approved the final manuscript.

## Supporting information



Figure S1. Mean children dietary diversity score (CDDS) in the intervention groups at baseline survey (2.37 ± 1.11), follow up at 3th months (3.56 ± 0.78), follow up at 4th months (3.69 ± 0.75), follow up at 5th months (3.84 ± 0.63), and at endline survey (3.87 ± 1.06).
