# Supplementary material for: Nutrition education in Southeast Sulawesi Province, Indonesia: A cluster randomized controlled study
Source: Matern Child Nutr. 2020 May 28;16(4):e13030. doi: 10.1111/mcn.13030 (PMC7507461; doi:10.1111/mcn.13030)
Supplement: Supplementary file 1 — Figure S1. Mean children dietary diversity score (CDDS) in the intervention groups at baseline survey (2.37 ± 1.11), follow up at 3th months (3.56 ± 0.78), follow up at 4th months (3.69 ± 0.75), follow up at 5th months (3.84 ± 0.63), and at endline survey (3.87 ± 1.06). [file MCN-16-e13030-s001.docx]

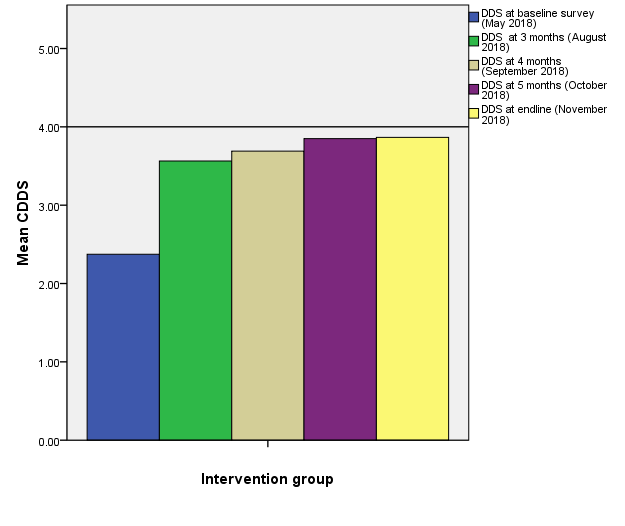


Supplementary figure 1. Mean children dietary diversity score (CDDS) in the intervention groups at baseline survey (2.37±1.11), follow up at 3th months (3.56±0.78), follow up at 4th months (3.69±0.75), follow up at 5th months (3.84±0.63), and at endline survey (3.87±1.06).
